# Improving Access to Preconception Care for Women with Severe and Enduring Mental Illness, Through Creation of a Perinatal MDT Clinic in a Rehabilitation Psychiatry Setting

**DOI:** 10.1192/bjo.2025.10337

**Published:** 2025-06-20

**Authors:** Elizabeth Ashley, Jessica Foster, Sarah Fitch, Katie Fergus, Amy Sheppard

**Affiliations:** Cardiff and Vale UHB, Cardiff, United Kingdom

## Abstract

**Aims:** Preconception planning is an essential component of improving maternal and child health, particularly for individuals with mental health conditions. Within this population those with severe and enduring mental illnesses face significant barriers to accessing preconception care and are at higher risk of unplanned pregnancy, leading to suboptimal outcomes for both mothers and babies. This poster outlines the establishment of a preconception planning clinic for individuals within a mental health rehabilitation setting, developed through a collaborative initiative between the Rehabilitation and Recovery Service, Perinatal Mental Health Service, All Wales Psychiatric Genomics Service and Sexual Health services.

The clinic aims to provide personalised, multidisciplinary support to women with severe and enduring mental health conditions who are of childbearing age, ensuring that their mental health, medical, and social needs are addressed in a holistic and coordinated manner. Key components include individualised care planning, medication review, counselling on genetic risk to the baby, and psychosocial support, as well as the provision of education on reproductive health, contraception, and healthy relationships. Risks and impact to both mother and baby will be central to all discussions.

**Methods:** Through close collaboration between the Rehabilitation and Recovery Service, Perinatal Mental Health Service, All Wales Psychiatric Genomics Service and Sexual Health services, the clinic will foster an integrated approach to care, promoting early intervention and prevention of adverse outcomes. This initiative also supports service users in navigating the complexities of mental health during pregnancy, enhancing their confidence in planning for a safe and supported conception.

**Results:** The hope is that this clinic will promote proactive discussion of reproduction and sexual health within a population that have historically been overlooked in this aspect and reduce associated stigma and inequity.

**Conclusion:** The poster will showcase the clinic’s design, key challenges encountered, strategies for team integration, and initial outcomes from service users, with the aim of providing a model for other settings seeking to improve preconception care for individuals with severe and enduring mental illness.

